# Central Neurophysiological Alterations in Dystrophic *mdx* Mice Correlate With Reduced Hippocampal Levels of the Endogenous NMDA Receptor Ligand D‐Aspartate

**DOI:** 10.1111/jnc.70223

**Published:** 2025-09-06

**Authors:** Francesca Mastrostefano, Martina Garofalo, Tommaso Nuzzo, Claudio Bruno, Francesco Errico, Alessandro Usiello, Maria Egle De Stefano

**Affiliations:** ^1^ Department of Biology and Biotechnologies “Charles Darwin” Sapienza University of Rome Rome Italy; ^2^ Department of Environmental, Biological and Pharmaceutical Sciences and Technologies Università Degli Studi Della Campania “Luigi Vanvitelli” Caserta Italy; ^3^ CEINGE Biotecnologie Avanzate Franco Salvatore Napoli Italy; ^4^ Centre of Translational and Experimental Myology IRCCS Istituto Giannina Gaslini Genoa Italy; ^5^ Department of Neurosciences, Rehabilitation, Ophthalmology, Genetics, Maternal and Child Health (DINOGMI) University of Genoa Genoa Italy; ^6^ Department of Agricultural Sciences University of Naples “Federico II” Portici Italy; ^7^ Center for Research in Neurobiology “Daniel Bovet” Sapienza University of Rome Rome Italy

**Keywords:** amino acids, D‐aspartate, Duchenne muscular dystrophy, hippocampus, neurological disorders, spinal cord

## Abstract

Patients with Duchenne muscular dystrophy (DMD) may experience neurobehavioral and cognitive concerns, including psychiatric symptoms, due to the absence of full‐length dystrophin (Dp427), frequently accompanied by deficiencies in shorter isoforms. The lack of dystrophin affects neurophysiological processes from the uterine phase, impacting neural circuitry in brain regions such as the prefrontal cortex, hippocampus, and cerebellum. This leads to reduced inhibitory GABAergic transmission and altered hippocampal glutamatergic signaling. The resulting imbalance between inhibitory and excitatory inputs contributes to the neurodevelopmental and cognitive deficits observed in DMD. Recent studies have reported correlations between serum levels of D‐aspartate and D‐serine, endogenous ligands of glutamatergic receptors, and conditions such as schizophrenia, spinal muscular atrophy, and aging. Furthermore, in a recent clinical study, we reported a general dysregulation of D−/L‐amino acids known to modulate glutamatergic neurotransmission in the serum of DMD patients, with significant correlations between muscle wasting, motor impairment, and alterations in L‐glutamate levels and the L‐glutamine/L‐glutamate ratio. To delve deeper into this matter, we conducted an extensive neurochemical analysis using high‐pressure liquid chromatography to measure the levels of the same D−/L‐amino acids across various brain regions, the spinal cord, and serum of the *mdx* mouse model of DMD. Our results revealed a significant reduction in prenatal D‐aspartate levels and postnatal levels of specific L‐amino acids in the hippocampus of dystrophic mice compared to wild type. In adult *mdx* mice, we also observed a near‐significant decrease in hippocampal D‐serine levels and a significant reduction in spinal cord D‐aspartate levels. This study provides the first evidence potentially linking D‐/L‐amino acid dysmetabolism in the hippocampus to the described neurophysiological alterations. Although further investigations are essential to validate this hypothesis, the mechanisms proposed here offer insight into how amino acid imbalances may contribute to the DMD‐associated neurological and cognitive deficits, thus supporting the rationale for developing future targeted therapeutic strategies.

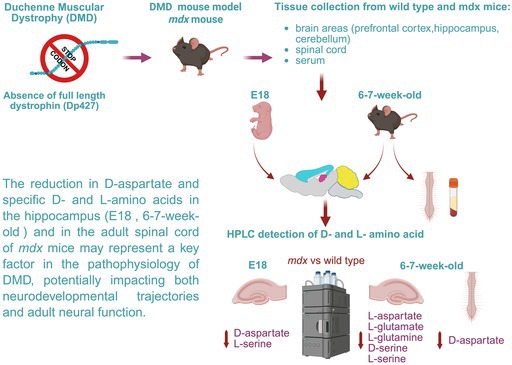

## Graphical abstract

Duchenne muscular dystrophy (DMD) is frequently associated with neurobehavioral and cognitive impairments, including psychiatric symptoms, resulting from the absence of full‐length dystrophin, alone or in combination with its shorter isoforms. This study provides new insights into how dystrophin deficiency may interfere with neurophysiological processes from developmental stages. By analyzing D‐ and L‐amino acid profiles in the brain, spinal cord, and serum of the *mdx* mouse model, we identified significant reductions in D‐aspartate and selected L‐amino acids in both the pre‐ and postnatal hippocampus, as well as in the adult spinal cord. These findings suggest a link between amino acid dysregulation and the neurophysiological abnormalities observed in DMD, potentially paving the way for targeted interventions to mitigate cognitive dysfunction. Image created with BioRender.com (www.biorender.com).

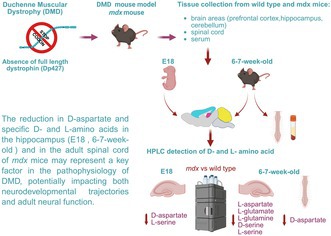

**Abbreviations**
AQP4: Aquaporin 4
CNS: Central nervous system
DAAO: D‐amino acid oxidase
D‐Asp/Total Asp: D‐aspartate/Total aspartate
DDO: D‐aspartate oxidase
DGC: Dystrophin‐associated glycoprotein complex
DMD: Duchenne muscular dystrophy
EAAT1: excitatory amino acid transporter 1
EAAT2: excitatory amino acid transporter 2
ECM: extracellular matrix
hDDO: human D‐aspartate oxidase
HPLC: high‐performance liquid chromatography
K_ir_4.1: potassium inward rectifier 4.1 channel
NMDA: N‐methyl‐D‐Aspartate
PFC: prefrontal cortex
PNS: peripheral nervous system
qRT‐PCR: quantitative reverse transcriptase polymerase chain reaction
RRID: research resource identifiers
SRR: serine racemase
TCA: tetrachloroacetic acid

## 1 Introduction

Duchenne muscular dystrophy (DMD) is an inherited, X‐linked neuromuscular disease characterized by progressive skeletal muscle wasting, primarily affecting limb and respiratory muscles (Muntoni et al. 2003; Duan et al. 2021; Ohlendieck and Swandulla 2021). However, significant morphological and functional alterations also impact cardiac (Klietsch et al. 1993) and smooth muscles (Hoffman et al. 1988; Halayko and Stelmack 2005; Manokaran et al. 2020), as well as other systems and apparatuses, making DMD a complex multi‐system disease. Among these, cardiomyopathies, vascular defects, and gastrointestinal dysfunction have been described (Muntoni et al. 2003; Lo Cascio et al. 2016; Duan et al. 2021; Dowling et al. 2021; Ohlendieck and Swandulla 2021).

Affecting approximately 1 in 5000 males (Crisafulli et al. 2020), the disease is caused by mutations in the large *DMD* gene located on the X chromosome (2.5 Mb of Xp21). The gene encodes a 427 kDa cytoskeletal protein known as dystrophin (Dp427), the expression of which is regulated by three promoters active in specific tissues/cells: the M promoter (skeletal and cardiac muscles), the P promoter (cerebellar Purkinje cells), and the B promoter (hippocampus, cortex) (Blake et al. 2002; Muntoni et al. 2003; Ohlendieck and Swandulla 2021). The *DMD* gene also encodes several short dystrophin isoforms transcribed from independent promoters located along the gene. These isoforms are expressed in various cell types, including specific neuronal and glial cell populations of the central (CNS) and peripheral/autonomic (PNS) nervous systems (De Stefano et al. 1997; Blake et al. 2002; Tozawa et al. 2012; Duan et al. 2021). The severity of the pathology depends on the type of mutation (out‐of‐frame or in‐frame) and its position along the gene, as these factors determine not only the loss of the full‐length dystrophin isoform, but also the number of additional isoforms affected.

In muscles, as in other cell types, dystrophin and its shorter isoforms bind to a large glycoprotein complex, the dystrophin‐associated glycoprotein complex (DGC), where they play crucial diversified roles. These include maintaining the integrity of the sarcolemma by bridging the cortical cytoskeleton to proteins of the extracellular matrix (ECM), as well as stabilizing various intracellular signaling molecules, membrane receptors, ion channels, and neurotransmitter receptors (Waite et al. 2012; Leyva‐Leyva et al. 2018; Davies and Nowak 2006; De Stefano et al. 2022; Dowling et al. 2021). In non‐muscle tissues, dystrophin isoforms associate with a DGC that is similar, but not identical, to the one found in muscle. The structural and functional properties of these complexes vary depending on the cell type, their level of differentiation or maturation, subcellular localization, and life stages (Waite et al. 2012; De Stefano et al. 2022).

DMD patients can exhibit various neurological conditions (Caspers Conway et al. 2015; reviewed in De Stefano et al. 2022; Hendriksen et al. 2024; Vaillend et al. 2025), highlighting neurobehavioral and neuropsychiatric aspects of the pathology. Clinical imaging has evidenced macroscopic brain abnormalities in the white matter of DMD patients, along with a decrease in both gray matter volume and cerebral blood flow (Doorenweerd et al. 2014; Preethish‐Kumar et al. 2020). In addition, several biochemical and metabolic alterations, including glucose hypometabolism, reduced lipid/protein peroxidation and catalase activity, and increased superoxide dismutase activity, have been reported in various brain regions (e.g., hippocampus, cortex, and cerebellum) (reviewed in De Stefano et al. 2022; Vaillend et al. 2025). In these areas, studies on autoptic human brains and *mdx* mice, well‐established animal models of the disease, have also evidenced fine structural and molecular alterations, likely due to the presence of a significant number of neurons and glial cells expressing Dp427 and its isoforms in normal individuals (Lidov et al. 1993; Lidov 1996; Doorenweerd et al. 2017; Huard and Tremblay 1992; Anderson et al. 2002; Briatore et al. 2020). Among these, the impact that Dp427 and its isoforms have on GABAergic and glutamatergic signaling in the CNS is particularly noteworthy. In this regard, a significant reduction of GABA_A_ receptor clusters on both cerebellar Purkinje cells and hippocampal neurons (reviewed in Vaillend et al. 2025; Knuesel et al. 1999; Fritschy et al. 2003; Craig and Kang 2007; Vaillend et al. 2010), as well as altered presynaptic organization of glutamatergic synapses (Miranda et al. 2011), has been observed. Impaired GABA function (Vaillend and Billard 2002) and/or reduced sensitivity of the glutamatergic N‐methyl‐D‐aspartate receptors (NMDARs) to magnesium block (Vaillend, Ungerer, et al. 1999) possibly lead to an imbalance in excitatory/inhibitory homeostasis, which results in impaired hippocampal and cerebellar long‐term depression and enhanced short‐term hippocampal plasticity. Interestingly, unlike the progressive deterioration observed in muscles, alterations in the nervous system are established during pre‐ and postnatal development, classifying this aspect of DMD as neurodevelopmental in origin (reviewed in De Stefano et al. 2022). Although these alterations were previously considered “non progressive,” recent studies have revealed worsening of spatial learning and long‐term memory and anxiety‐related behavior in aging *mdx* mice (Bagdatlioglu et al. 2020).

In recent years, several studies have highlighted the role of two amino acids in the atypical D‐configuration, D‐serine and D‐aspartate, in the modulation of glutamatergic transmission. D‐serine and D‐aspartate act as co‐agonists and agonists of NMDARs, respectively (reviewed in Souza et al. 2023; Coyle et al. 2020; Errico et al. 2020). Furthermore, D‐aspartate activates metabotropic glutamate receptor 5 (mGluR5) in brain slices from neonatal rats (Molinaro et al. 2010). High levels of D‐aspartate are present very early in prenatal development across different regions of both human and rodent brains, sharply decreasing within days after birth (Dunlop et al. 1986; Neidle and Dunlop 1990; Wolosker et al. 2000). D‐serine is also highly enriched, but, differently from D‐aspartate, its levels remain elevated also in the adult brain (Hashimoto et al. 1993, 1995). Notably, the areas particularly enriched in D‐aspartate (reviewed in Errico et al. 2015) are among those most affected in DMD, such as the cortex, hippocampus, and cerebellum. Increasing evidence on the role of these D‐amino acids in the development of specific brain circuits (reviewed in Errico et al. 2015) has raised the question of whether some development‐associated neuropathologies could be influenced by imbalances in their levels (Usiello et al. 2020; Errico et al. 2020; Ling et al. 2023). Indeed, dysregulation of D‐aspartate and D‐serine metabolism has been reported in the prefrontal cortex of schizophrenia patients (Nuzzo et al. 2017; De Rosa et al. 2022; Errico et al. 2013; Balu and Coyle 2015) and is associated with cognitive decline in frail elderly individuals (Imarisio et al. 2024). Similarly, dysregulation of glutamate and serine metabolism has been observed in the cerebrospinal fluid of patients with severe spinal muscular atrophy (SMA), including decreased levels of L‐glutamate resulting from dysregulated L‐glutamine‐to‐L‐glutamate conversion (Hassan et al. 2024).

Recently, to identify specific biomarkers of the disease, levels of D‐ and L‐amino acids involved in glutamatergic receptor activation (e.g., L‐glutamate, L‐aspartate, D‐aspartate, glycine, and D‐serine) and their precursors (e.g., L‐glutamine, L‐asparagine, and L‐serine) were investigated in the serum of a cohort of DMD patients compared to age‐matched controls using High‐Performance Liquid Chromatography (HPLC) (Garofalo et al. 2025). The results have demonstrated dysregulation in the levels of several amino acids, with a particular reduction in L‐glutamate and the L‐glutamine/L‐glutamate ratio in dystrophic patients compared to controls. These alterations were directly correlated with muscle wasting and motor impairment but not with the neurological scores of the patients.

Building on this prior clinical knowledge, the present exploratory study aimed to investigate potential alterations in the levels of D‐ and L‐amino acids involved in glutamatergic neurotransmission across the prefrontal cortex, hippocampus, cerebellum, and spinal cord, as well as in serum, of both prenatal and adult *mdx* mice.

## 2 Materials and Methods

### 2.1 Animals

**FIGURE 1:**
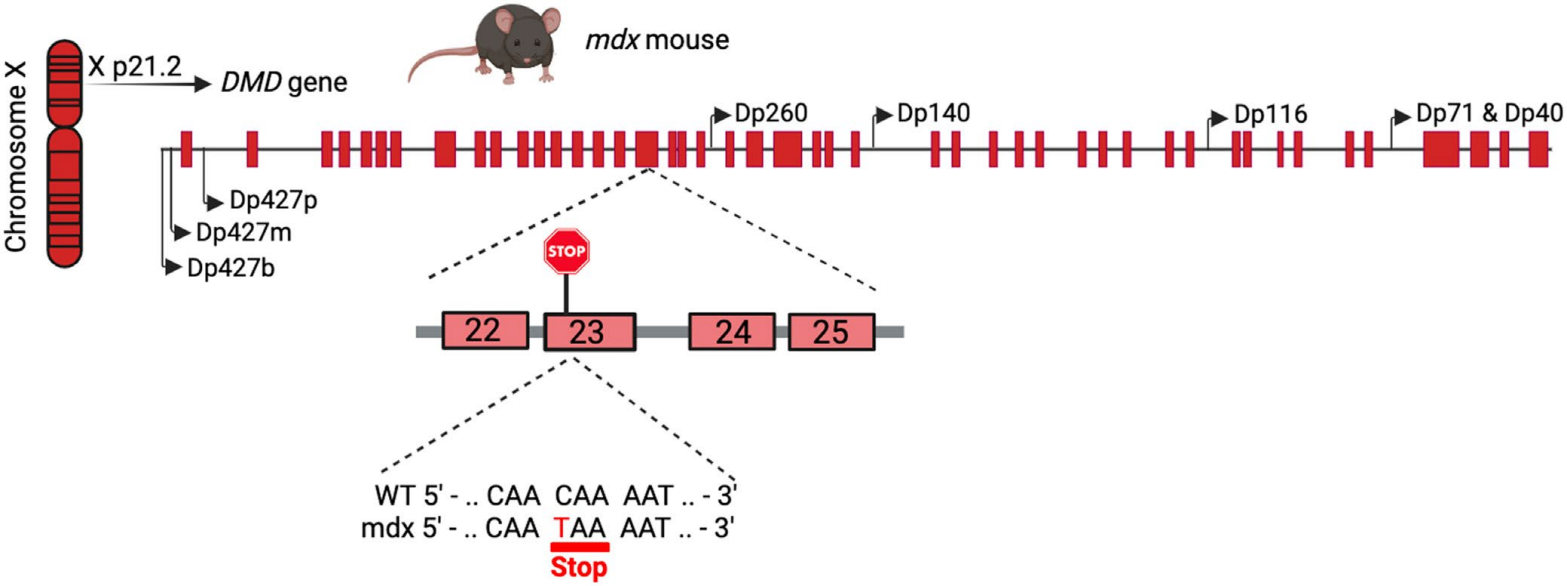
Diagram of the *mdx* mouse genetic model. The *mdx* mouse model features a point mutation in exon 23, leading to an early stop codon. This mutation prevents the expression of full‐length dystrophin (Dp427) but allows the expression of shorter dystrophin isoforms, which are produced from internal promoters. Image created with BioRender.com (www.biorender.com).

For this study, wild‐type (C57BL/10ScSnJ; RRID:IMSR_JAX:000476) and genetically dystrophic *mdx* (C57BL/10ScSn‐Dmdmdx/J; RRID:IMSR_JAX:001801) mice at different pre‐ and postnatal ages were used. Breeding pairs of both genotypes were originally obtained from The Jackson Laboratory (Bar Harbor, ME, USA) and were used to establish permanent colonies in our departmental animal facility. Consequently, all animals used in this study were born and raised in‐house. The *mdx* mouse model, commonly studied for DMD, has a natural nonsense mutation (C‐to‐T transition) in exon 23 of the *Dmd* gene, located on the X chromosome, that halts full‐length dystrophin expression while preserving short dystrophin isoforms (Sicinski et al. 1989) (Figure 1). This model exhibits the typical muscular degeneration, albeit milder than in humans because it occurs in definite time windows, where degenerative processes are followed by significant regeneration (Coulton, Curtin, et al. 1988; Coulton, Morgan, et al. 1988; Zaynitdinova et al. 2021), along with diverse brain and autonomic nervous system abnormalities. Although all *mdx* mice carry the mutation, genotypic controls are randomly performed to ensure the maintenance of the dystrophic mutation. Furthermore, for both wild‐type and *mdx* mice, matings never occur between closely related individuals, the male breeder changes for each litter, and both males and females are used for 3–4 L. Mice were housed in cages (four per cage) at a constant temperature of 22°C, maintained on a 12‐h light–dark cycle (lights on at 7 am), with free access to food and water. All studies complied with The Code of Ethics of the EU Directive 2010/63/EU. Efforts were made to minimize animal suffering, reduce the number of animals used, and utilize alternatives to in vivo techniques. Experimental procedures and protocols were approved by the Ethical Committee for Animal Research of the Italian Ministry of Public Health (n° 44/2023‐PR).

### 2.2 HPLC Analysis of Amino Acid Content

For the HPLC analysis, we used a total of 20 wild‐type (wt) and 20 *mdx* mice, with an initial aim of 10 mice per genotype and age group. The number of samples yielding analyzable results was as follows: 9 wt and 9 *mdx* for E18 brains; 7 wt and 7 *mdx* for adult mouse brains; 8 wt and 9 *mdx* for adult spinal cords; and 9 wt and 10 *mdx* for adult mouse serum. Sample size was determined based on previous studies (Punzo et al. 2016; De Rosa et al. 2020). Mice were deeply anesthetized by inhalation of 3% isoflurane (Merial, Milan, Italy) and euthanized by decapitation. Levels of D‐ and L‐amino acids in serum and brain homogenates of hippocampus, cerebellum, prefrontal cortex, and spinal cord were analyzed by HPLC, as previously described (De Rosa et al. 2020; Imarisio et al. 2024) with minor modifications. Serum samples were mixed in a 1:10 dilution with HPLC‐grade methanol (Sigma‐Aldrich, 34 860) and centrifuged at 13 000 *g* for 10 min; supernatants were dried and then suspended in 0.2 M TCA. Brain samples were homogenized in 1:10 (w/v) 0.2 M trichloroacetic acid (TCA), sonicated (3 cycles, 10s/cycle), and centrifuged at 13 000 *g* for 20 min. Pellets were stored at −80°C for protein quantification. TCA (Sigma‐Aldrich, T6399) supernatants from serum and tissue samples were then neutralized with 0.2 M NaOH (Sigma‐Aldrich, 106 469) and subjected to precolumn derivatization with o‐phthaldialdehyde (Sigma‐Aldrich, P0657)/N‐acetyl‐L‐cysteine (Sigma‐Aldrich, A7250) in 50% methanol. Diastereoisomer derivatives were resolved on a Symmetry C8 5‐μm reversed‐phase column (Waters, 4.6 × 250 mm). Identification and quantification of the amino acids were based on retention times and peak areas compared with those associated with external standards. The identity of the D‐aspartate was further validated by selective degradation using recombinant human D‐aspartate oxidase (hDDO) (Katane et al. 2017, 2018). Specifically, 12.5 μg of hDDO were added to the samples, incubated at 30°C for 3 h, and then derivatized. For brain tissue samples, total protein content was determined using the Bradford assay (Biorad, 500‐0006) method after resolubilizing the TCA‐precipitated protein pellets

Amino acid level in the serum samples was expressed as micromolar (μM). The concentration of amino acids in tissue homogenates was normalized to the total protein content and expressed as nmol/mg protein.

### 2.3 Quantitative Reverse Transcriptase Polymerase Chain Reaction (qRT‐PCR)

For this study, we used embryonic day 18 (E18), postnatal day 10 (P10), and male young adult (6–7‐week‐old) wild‐type and dystrophic *mdx* mice (*n* = 6/age/genotype). Mice were deeply anesthetized by inhalation of 3% isoflurane (Merial, Milan, Italy) and euthanized by decapitation. The prefrontal cortex (PFC), hippocampus, and cerebellum of E18, P10, and 6‐ to 7‐week‐old wild‐type and *mdx* mice were dissected within 20 s on an ice‐cold surface. All tissue samples were pulverized in liquid nitrogen and stored at −80°C until use. Total RNA was extracted using the RNeasy mini kit (QIAGEN Cat. No. 74106) following the manufacturer's instructions (Querques et al. 2015). RNA integrity was assessed using denaturing agarose gel electrophoresis (presence of sharp 28S, 18S, and 5S bands) and spectrophotometry (Nano‐Drop 2000, ThermoFisher Scientific, Waltham, MA, USA). RNA was purified to eliminate contaminating genomic DNA using recombinant DNase (QIAGEN, Cat. No. 74106). One microgram of total RNA from each sample was reverse transcribed with the Quanti Tect Reverse Transcription (QIAGEN, Cat. No. 205313) using oligo‐dT and random primers according to the manufacturer's instructions. qRT‐PCR amplifications were performed using the LightCycler 480 SYBR Green I Master (Roche Diagnostic, Cat. No. RO04707516001) in a LightCycler 480 Real‐Time thermocycler. Reaction conditions were 10 s at 95°C for initial denaturation, followed by 40 cycles of 10 s at 95°C for denaturation, 10 s at 60°C for annealing, and 6 s at 72°C for elongation. Relative gene expression was quantified by the ΔΔCt. mRNA levels of *Ddo* (D‐aspartate oxidase), *Srr* (Serine racemase), and *Daao* (D‐amino acid oxidase) were detected. *b‐actin* and *Ppp1a* mRNA levels were used as internal references, and each experiment was replicated three times. The primers used are listed in Table S1.

### 2.4 Statistical Analysis

Normality distribution was assessed by q‐q plot and the Shapiro–Wilk test. Since the data did not always follow a normal distribution, a comparison between wild‐type and *mdx* mice was performed with the non‐parametric Mann–Whitney *U* test (HPLC). Comparisons among more than two groups were evaluated by two‐way ANOVA followed by Fisher's post hoc analysis between genotypes (qRT‐PCR). Data were analyzed by using the Prism GraphPad 10 software and expressed as median with interquartile range (IQR). Outlier analysis was not performed, and all data were included in the analysis. Sample sizes for each experiment are indicated in the respective figure legends. All differences were considered statistically significant for *p* ≤ 0.05.

## 3 Results

### 3.1 D‐ and L‐Amino Acids Are Differently Modulated in the Hippocampus of E18 and 6 to 7‐Week‐Old *mdx* Mice Compared to Wild Type Mice

**FIGURE 2:**
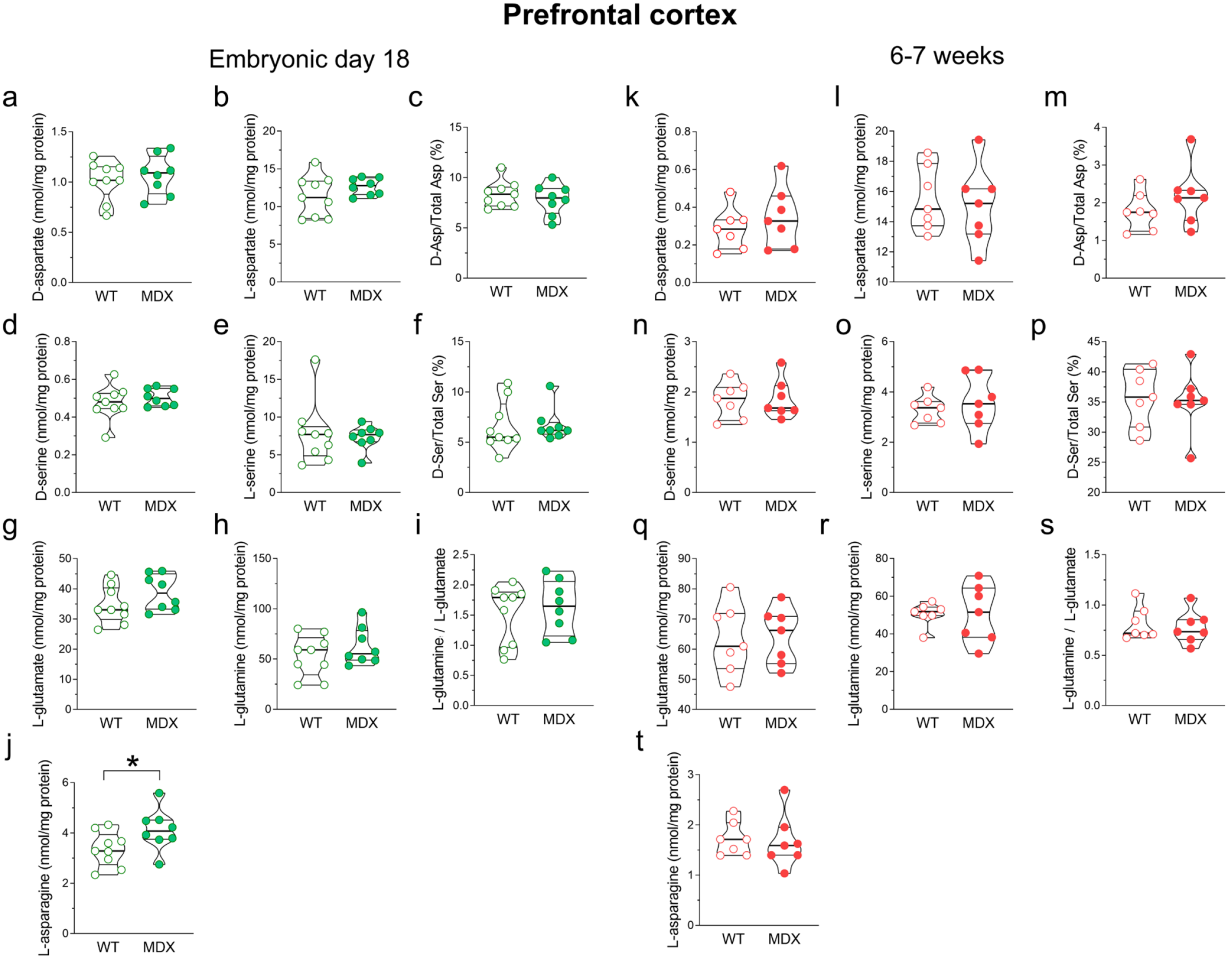
Amino acid levels in the prefrontal cortex of E18 and of 6–7‐week‐old wild‐type and *mdx* mice. HPLC analysis of (a, k) D‐aspartate, (b, l) L‐aspartate, (c, m) D‐Asp/Total Asp, (d, n) D‐serine, (e, o) L‐serine, (f, p) D‐Ser/Total D‐Ser, (g, q) L‐glutamate, (h, r) L‐glutamine, (i, s) L‐glutamine/L‐glutamate, (j, t) L‐asparagine (E18 mice, *n* = 9/genotype; 6–7‐week‐old mice, *n* = 7/genotype). Amino acid content is expressed as nmol/mg protein, whereas D‐Asp/Total Asp and D‐Ser/Total Ser ratios are expressed as percentages. In the prefrontal cortex of E18 *mdx* mice, L‐asparagine levels are significantly higher compared to wild‐type mice (j). In adult mice, no significant differences are observed in any of the analyzed amino acids between the two genotypes. Data are represented as violin box plots showing median, interquartile range (IQR), minimum, and maximum and analyzed by the Mann–Whitney *U* test. *p < 0.05.

**FIGURE 3:**
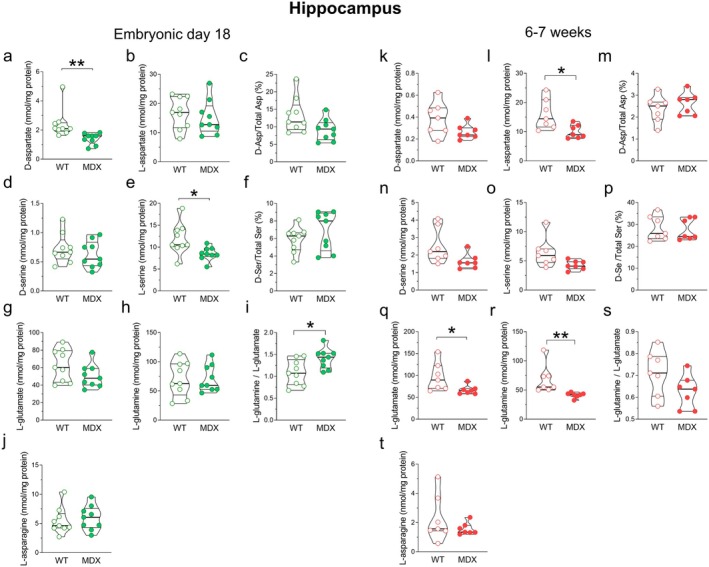
Amino acid levels in the hippocampus of E18 and 6–7‐week‐old wild‐type and *mdx* mice. HPLC analysis of (a, k) D‐aspartate, (b, l) L‐aspartate, (c, m) D‐Asp/Total Asp, (d, n) D‐serine, (e, o) L‐serine, (f, p) D‐Ser/Total D‐Ser, (g, q) L‐glutamate, (h, r) L‐glutamine, (i, s) L‐glutamine/L‐glutamate, (j, t) L‐asparagine (E18 mice, *n* = 9/genotype; 6–7‐week‐old mice, *n* = 7/genotype). Amino acid content is expressed as nmol/mg protein, whereas D‐Asp/Total Asp and D‐Ser/Total Ser ratios are expressed as percentages. In the hippocampus of E18 *mdx* mice, levels of D‐aspartate (a) and L‐serine (e) are significantly lower compared to wild‐type mice. The D‐aspartate to total aspartate (D‐Asp/Total Asp) ratio shows a trend toward decrease (*p* = 0.062), while the L‐glutamine/L‐glutamate ratio is significantly higher in *mdx* mice compared to wild type. In adult mice, D‐aspartate levels in the dystrophic genotype tend to remain lower compared to wild‐type mice, although this difference is not significant (k). Significantly reduced levels are, instead, observed for L‐aspartate (l), L‐glutamate (q), and L‐glutamine (r). Additionally, D‐serine (*p* = 0.073) and L‐serine (*p* = 0.053) levels are near statistical significance in *mdx* mouse samples compared to wild type. Data are represented as violin box plots showing median, interquartile range (IQR), minimum, and maximum and analyzed by the Mann–Whitney *U* test. *p < 0.05; **p < 0.01.

**FIGURE 4:**
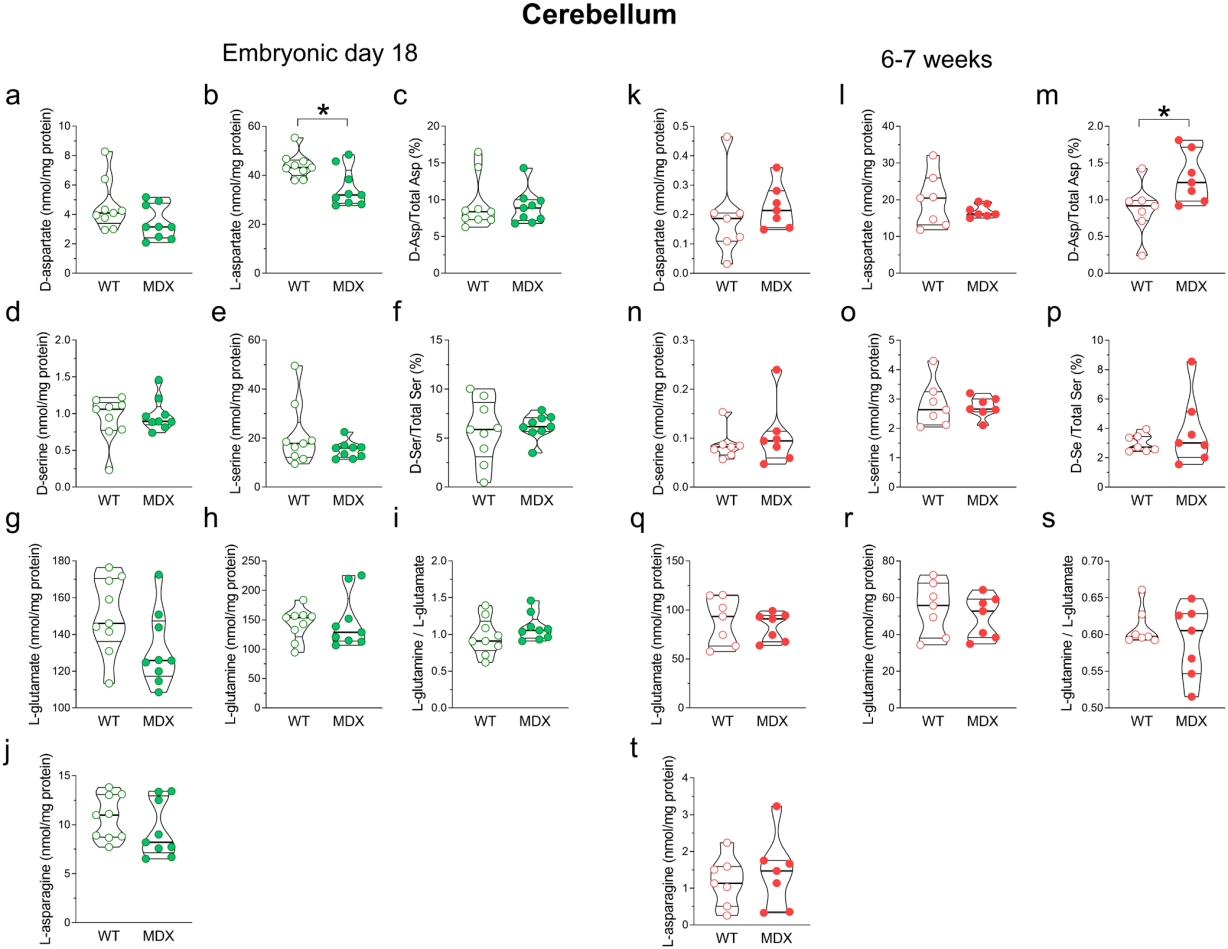
Amino acid levels in the cerebellum of E18 and 6–7‐week‐old wild‐type and *mdx* mice. HPLC analysis of (a, k) D‐aspartate, (b, l) L‐aspartate, (c, m) D‐Asp/Total Asp, (d, n) D‐serine, (e, o) L‐serine, (f, p) D‐Ser/Total D‐Ser, (g, q) L‐glutamate, (h, r) L‐glutamine, (i, s) L‐glutamine/L‐glutamate, (j, t) L‐asparagine (E18 mice, *n* = 9/genotype; 6–7‐week‐old mice, *n* = 7/genotype). Amino acid content is expressed as nmol/mg protein, whereas D‐Asp/Total Asp and D‐Ser/Total Ser ratios are expressed as percentages. HPLC analysis of (a, k) D‐aspartate, (b, l) L‐aspartate, (c, m) D‐Asp/Total Asp, (d, n) D‐serine, (e, o) L‐serine, (f, p) D‐Ser/Total D‐Ser, (g, q) L‐glutamate, (h, r) L‐glutamine, (i, s) L‐glutamine/L‐glutamate, (j, t) L‐asparagine (E18 mice, *n* = 9/genotype; 6–7‐week‐old mice, *n* = 7/genotype). Amino acid content is expressed as nmol/mg protein, whereas D‐Asp/Total Asp and D‐Ser/Total Ser ratios are expressed as percentages. HPLC analysis of (a, k) D‐aspartate, (b, l) L‐aspartate, (c, m) D‐Asp/Total Asp, (d, n) D‐serine, (e, o) L‐serine, (f, p) D‐Ser/Total D‐Ser, (g, q) L‐glutamate, (h, r) L‐glutamine, (i, s) L‐glutamine/L‐glutamate, (j, t) L‐asparagine (E18 mice, *n* = 9/genotype; 6–7‐week‐old mice, *n* = 7/genotype). Amino acid content is expressed as nmol/mg protein, whereas D‐Asp/Total Asp and D‐Ser/Total Ser ratios are expressed as percentages. In the cerebellum of E18 *mdx* mice, only levels of L‐aspartate are significantly lower compared to wild‐type mice (b), while L‐glutamate levels show a trend toward decrease (*p* = 0.09) (g). In 6–7‐week‐old mice, no significant differences are observed between the two genotypes in any of the analyzed D‐ and L‐amino acids, except for a higher D‐aspartate to total aspartate (D‐Asp/Total Asp) ratio in *mdx* mice compared to wild type. Data are represented as violin box plots showing median, interquartile range (IQR), minimum, and maximum and analyzed by the Mann–Whitney *U* test. *p < 0.05.

Levels of D‐aspartate and other neuroactive D‐ and L‐amino acids, such as L‐aspartate, L‐glutamate, L‐glutamine, D‐serine, L‐serine, L‐asparagine, D‐aspartate/total aspartate (D‐Asp/Total Asp), and D‐serine/total serine (D‐Ser/Total Ser), were measured by HPLC in the brains of E18 and 6‐ to 7‐week‐old *mdx* and wild‐type mice (Figures 2, 3, 4). In particular, we focused our study on tissue homogenates of PFC, hippocampus, and cerebellum that are known to be affected in DMD (reviewed in De Stefano et al. 2022).

HPLC analysis of PFC homogenates from both E18 and 6‐ to 7‐week‐old mice revealed no significant differences in the analyzed amino acids, except for L‐asparagine, which was significantly higher (Mann–Whitney *U* = 14; *p* = 0.036; Figure 2j) in E18 *mdx* mice compared to wild type.

Conversely, in the hippocampus we reported significant differences in the levels of several of the analyzed amino acids (Figure 3). Notably, the D‐aspartate content was significantly lower in E18 *mdx* mice compared to the wild‐type (Mann–Whitney *U* = 3; *p* = 0.003; Figure 2a), and, based on this change, the D‐Asp/Total Asp ratio showed a decrease close to significance (*p* = 0.062; Figure 2c). In line with the reduction in D‐aspartate, E18 *mdx* mice also exhibited significantly lower levels of L‐serine (the precursor of D‐serine) (Mann–Whitney *U* = 12; *p* = 0.0106; Figure 3e) compared to wild‐type mice. We also found a slight, though not significant, decrease in L‐glutamate levels (Figure 3g). Consequently, the L‐glutamine/L‐glutamate ratio was significantly higher in the hippocampus of E18 *mdx* mice compared to age‐matched wild‐type mice (Mann–Whitney *U* = 15; *p* = 0.0244; Figure 3i).

In the hippocampus of young adult (6–7 weeks) *mdx* mice, significantly lower levels of L‐aspartate (Mann–Whitney *U* = 6; *p* = 0.0175; Figure 3l) were observed in *mdx* mice compared to the wild‐type mice. *mdx* mice also displayed reduced levels of D‐serine (Mann–Whitney *U* = 10; *p* = 0.073; Figure 3n) and its precursor L‐serine (Mann–Whitney *U* = 9; *p* = 0.053; Figure 3o), both nearing statistical significances. Finally, decreased levels of L‐glutamate (Mann–Whitney *U* = 9; *p* = 0.017; Figure 3q) and L‐glutamine (Mann–Whitney *U* = 0; *p* = 0.0006; Figure 3r), a common precursor of both GABA and glutamate, were also observed in the dystrophic phenotype compared to control.

HPLC analysis of cerebellar homogenates from E18 mice revealed levels of L‐aspartate in *mdx* mice lower than in wild‐type mice (Mann–Whitney *U* = 17; *p* = 0.04; Figure 4b). No significant differences were observed for any of the analyzed amino acids between adult wild‐type and *mdx* mice, except for a trend toward decreased levels of L‐aspartate, which makes the D‐Asp/Total Asp ratio significantly higher (Mann–Whitney *U* = 8; *p* = 0.038) in the *mdx* mice compared to wild‐type mice (Figure 4m).

### 3.2 D‐Aspartate Levels Are Significantly Lower in the Spinal Cord of Adult *mdx* Mice Compared to Wild Type

Sensory motor control of skeletal muscles is mediated through the spinal cord. Sensory neurons, which connect to muscles via long peripheral fibers, transmit information that enters via the dorsal root and are differentially distributed to the dorsal and ventral horns, where motoneurons are located. This establishes a closed loop that coordinates muscle tone and contraction.

**FIGURE 5:**
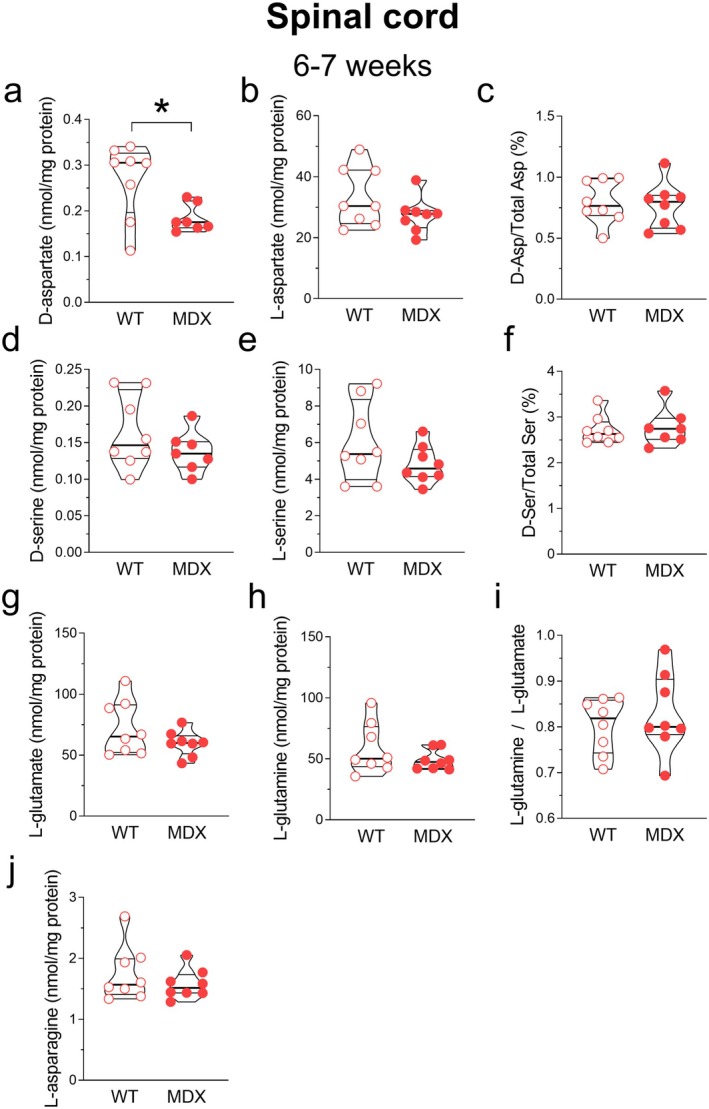
Amino acid levels in the lumbar spinal cord of 6–7‐week‐old wild‐type and *mdx* mice. HPLC analysis of (a) D‐aspartate, (b) L‐aspartate, (c) D‐Asp/Total D‐Asp, (d) D‐serine, (e) L‐serine, (f) D‐Ser/Total D‐Ser, (g) L‐glutamate, (h) L‐glutamine, (i) L‐glutamine/L‐glutamate, (j) L‐asparagine (wild type, *n* = 8; *mdx* mice, *n* = 7–8). Amino acidic content is expressed as nmol/mg protein, whereas D‐Asp/Total Asp and D‐Ser/Total Ser ratios are expressed as percentages. Levels of D‐aspartate in the lumbar spinal cord homogenates from 6 to 7‐week‐old *mdx* mice are significantly lower compared to wild type (a). Data are represented as violin box plots showing median, interquartile range (IQR), minimum, and maximum and analyzed by the Mann–Whitney *U* test. *p < 0.05.

Previous studies on patients with DMD have shown altered motor unit firing rates, which reflect altered functional properties of motoneurons (Piotrkewicz 1999; Piotrkewicz et al. 1999). Based on these findings, we analyzed whether 6‐ to 7‐week‐old *mdx* mice, undergoing the first cycle of skeletal muscle degeneration, exhibit changes in amino acid levels within the lumbar tract of the spinal cord. Our results demonstrated significantly lower levels of the sole D‐aspartate in *mdx* mice compared to wild‐type mice (Mann–Whitney *U* = 9; *p* = 0.029; Figure 5a).

### 3.3 Amino Acids Profile in the Serum of Adult *mdx* Mice Is Unaltered Compared to Wild Type

**FIGURE 6:**
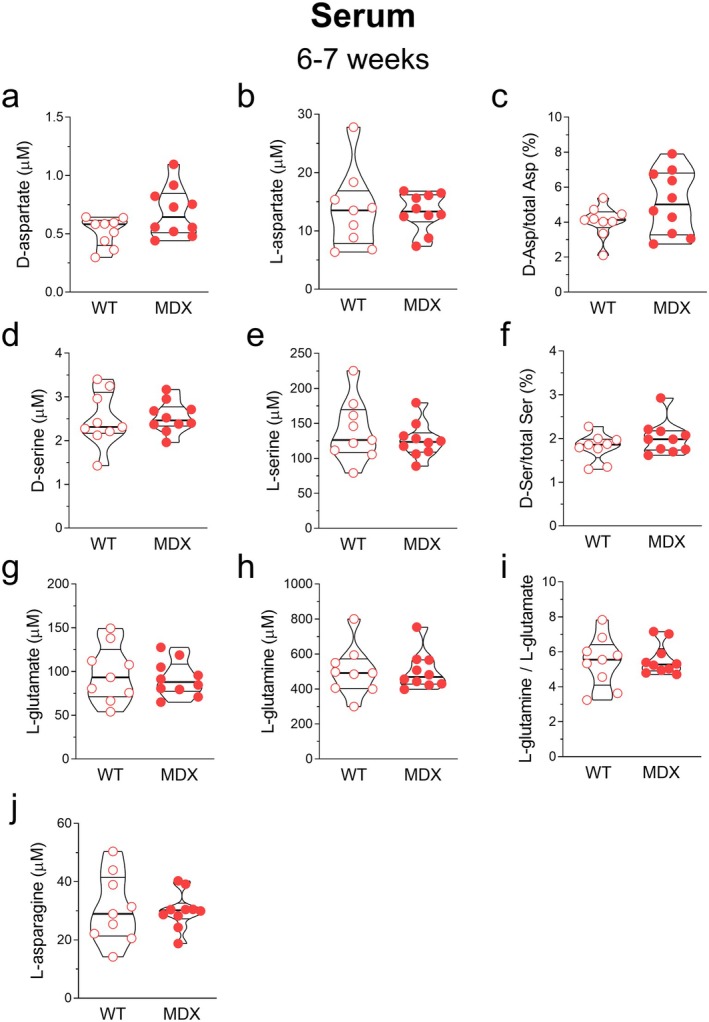
Amino acid levels in the serum of 6–7‐week‐old wild‐type and *mdx* mice. HPLC analysis of (a) D‐aspartate, (b) L‐aspartate, (c) D‐Asp/Total D‐Asp, (d) D‐serine, (e) L‐serine, (f) D‐Ser/Total D‐Ser, (g) L‐glutamate, (h) L‐glutamine, (i) L‐glutamine/L‐glutamate, and (j) L‐asparagine (wild‐type mice, *n* = 9; *mdx* mice, *n* = 10). Amino acid content is expressed as nmol/mg protein, whereas D‐Asp/Total Asp and D‐Ser/Total Ser ratios are expressed as percentages. No significant differences are observed in any of the analyzed D‐ and L‐amino acids between the two genotypes. Data are expressed as mean ± SEM and analyzed by the Mann–Whitney *U* test.

As previously mentioned, a recent study by Garofalo et al. (2024) demonstrated a significant alteration in the levels of the L‐glutamate and L‐glutamine/L‐glutamate ratio in the serum of dystrophic patients compared to controls, with a direct correlation to muscle wasting and motor impairment. In contrast, changes in these amino acid profiles did not correlate with the neurological score of the dystrophic patients, suggesting that these modifications were strictly related to muscle wasting rather than to neurological impairment. In this study, we replicated this analysis on the blood serum of 6–7‐week‐old *mdx* mice, fully aware that the dystrophic *mdx* genotype, although exhibiting the typical pattern of skeletal muscle fiber degeneration associated with DMD (Coulton, Curtin, et al. 1988; Coulton, Morgan, et al. 1988), displays significantly milder motor symptoms compared to humans. The results clearly showed that, unlike in human patients, the levels of all D‐ and L‐amino acids analyzed, including the D‐Asp/Total Asp and D‐Ser/Total Ser ratios, did not differ significantly between the two genotypes (Figure 6).

### 3.4 The Gene Expression of the Enzymes Involved in D‐Amino Acid Metabolism Is Unaltered in Cerebral Areas of *mdx* Mice

We analyzed whether the changes in cerebral D‐amino acid levels might result from imbalanced expression of the genes encoding the key enzymes responsible for their synthesis and degradation, such as D‐aspartate oxidase (DDO), D‐amino acid oxidase (DAO), and serine racemase (SRR). DDO and DAO are the catabolic enzymes responsible for D‐aspartate and D‐serine degradation, respectively (Molla et al. 2020; Pollegioni et al. 2018, 2020). On the other hand, SRR is the enzyme responsible for D‐serine biosynthesis (Wolosker et al. 1999). We performed qRT‐PCR to analyze the mRNA expression levels of *Ddo*, *Srr*, and *Daao* in the hippocampus, PFC, and cerebellum of *mdx* and wild‐type mice at E18, P10, and 6–7 weeks.

In line with its physiological expression (Punzo et al. 2016), the *Ddo* mRNA levels progressively increased from E18 to adulthood in both wild‐type and *mdx* mice (two‐way ANOVA, age effect; PFC: *F*_(2,30)_ = 153.6, *p* < 0.0001; hippocampus: *F*_(2,30)_ = 112.0, *p* < 0.0001; cerebellum: *F*_(2,30)_ = 762.4, *p* < 0.0001; Figure S1a,d,g), but no significant differences emerged between the two genotypes (two‐way ANOVA, age *x* genotype interaction; PFC: *F*_(2,30)_ = 1.590, *p* = 0.2206; hippocampus: *F*_(2,30)_ = 1.184, *p* = 0.3201; cerebellum: *F*_(2,30)_ = 0.3750, *p* = 0.6905; Figure S1a,d,g). Similarly, mRNA levels of *Srr* increased in all three brain regions and in both genotypes over time (two‐way ANOVA, age effect; PFC: *F*_(2,30)_ = 114.6, *p* < 0.0001; hippocampus: *F*_(2,30)_ = 79.56, *p* < 0.0001; cerebellum: *F*_(2,30)_ = 77.86, *p* < 0.0001; Figure S1b,e,h), again with no significant differences between *mdx* and wild‐type mice (two‐way ANOVA, age *x* genotype interaction; PFC: *F*_(2,30)_ = 0.4909, *p* = 0.6169; hippocampus: *F*_(2,30)_ = 0.1680, *p* = 0.8461; cerebellum: *F*_(2,30)_ = 0.0034, *p* = 0.9966; Figure S1b,e,h).

Detectable mRNA levels of *Daao* were found only in cerebellar homogenates. In both genotypes, *Daao* mRNA expression was low at E18 and P10 but increased sharply in adult mice (two‐way ANOVA, age effect; *F*_(2,30)_ = 2.871, *p* < 0.0001; Figure S1i). Although no significant differences were observed between the two genotypes, mRNA levels were slightly higher in *mdx* mice than in the wild‐type mice at 6–7 weeks of age (two‐way ANOVA, age *x* genotype interaction; *F*_(2,30)_ = 2.871, *p* = 0.0723; Figure S1).

## 4 Discussion

As outlined in the introduction, the present observational study originates from our previous clinical investigation, which represents the conceptual and methodological foundation of the current work. In that study, we employed HPLC to analyze the serum of a well‐characterized cohort of young DMD patients, identifying significant alterations in several D‐ and L‐amino acids, including a marked reduction in L‐glutamate levels and the L‐glutamine/L‐glutamate ratio (Garofalo et al. 2025). Starting from these systemic findings, the current study aimed to determine whether similar or more localized alterations might occur within the CNS. To this end, we extended our analysis to specific brain regions and the spinal cord, alongside serum, using the *mdx* mouse model of DMD. This approach enabled a more in‐depth investigation of the neurochemical landscape underlying the disease. Our findings confirm a dysregulation of amino acid homeostasis and, for the first time, reveal that levels of D‐aspartate, L‐aspartate, and other D‐/L‐amino acids critically involved in NMDAR‐related CNS development and function, including D‐ and L‐serine, undergo age‐ and region‐specific changes in the CNS of *mdx* mice. Notably, among the three brain regions examined—prefrontal cortex, hippocampus, and cerebellum, which are consistently reported as being the most affected in DMD, although to varying degrees—the hippocampus emerged as the most severely impacted, both in prenatal (E18) and young adult (6–7 weeks) *mdx* mice. In addition, we detected abnormal levels of D‐aspartate in the adult lumbar spinal cord, a region that hosts motoneurons innervating the hindlimbs and key connections of the sensory‐motor circuitry.

These results can be discussed by moving on to two different grounds. The first is that the absence of Dp427 alone, characteristic of the *mdx* mouse model of DMD, once again proves to have a significant impact on aspects related to the dynamic phases of neurodevelopment during the prenatal stage, with potential consequences extending into adult life as well. As observed in both patients and animal models of DMD, the described cognitive and neurological disturbances are accompanied by corresponding morphological, circuital, cellular, and molecular alterations typically arising during pre‐ and postnatal neurodevelopment (Licursi et al. 2012; Lombardi et al. 2017; Fragapane et al. 2020; Persiconi et al. 2020; reviewed in De Stefano et al. 2022). This highlights the in utero phase as a critical period in the development of functional and physiological alterations that could, over time, further impact the quality of life of DMD patients. It is during the prenatal phase that D‐aspartate and D‐serine play a predominant role. D‐aspartate is highly enriched during brain development and drastically declines after birth (Dunlop et al. 1986; Hashimoto et al. 1993, 1995; Sakai et al. 1998; Punzo et al. 2016; De Rosa et al. 2020), while D‐serine follows a different pattern, steadily increasing during prenatal stages and after birth and reaching plateau levels in adolescence (Hashimoto et al. 1993). Although present in several areas of the CNS, both D‐amino acids are particularly evident in those regions recognized among the most damaged in DMD, namely the hippocampus, prefrontal cortex, and cerebellum (Wolosker et al. 2000, 2008; Errico et al. 2009, 2015).

According to our data, a significant imbalance in the levels of D‐aspartate, D‐serine, and specific L‐amino acids was primarily observed in the hippocampus, while the prefrontal cortex and cerebellum appeared to be unaffected. At the prenatal stage E18, *mdx* mice displayed significantly lower levels of D‐aspartate and L‐serine compared to wild type. At the same stage, we also observed a significantly higher L‐glutamine/L‐glutamate ratio, likely reflecting an overall decrease, although not significant, in the L‐glutamate levels. In addition, 6–7‐week‐old *mdx* mice were characterized by a significant reduction in L‐aspartate, L‐glutamate, and L‐glutamine compared to wild‐type mice. These data strongly suggest that the alteration of the glutamatergic signaling system in this region, well described in adult *mdx* mice (Vaillend, Ungerer, et al. 1999; Vaillend, Billard, et al. 1999; Vaillend and Billard 2002; Miranda et al. 2011), might begin during the pre‐ and early postnatal stages. Indeed, D‐aspartate is an endogenous agonist of both NMDARs (Fagg and Matus 1984; Olverman et al. 1988; Errico, Nistico, et al. 2008; Errico, Rossi, et al. 2008; Errico, Nistico, Napolitano, Oliva, et al. 2011) and mGluR5 (Molinaro et al. 2010), and its excitatory role during brain development (Errico et al. 2012; Punzo et al. 2016; Krashia et al. 2016; Sacchi et al. 2017) may be particularly relevant, considering that alterations in NMDAR activity have been reported in *mdx* mice (Vaillend, Ungerer, et al. 1999).

Studies conducted on a *Ddo* model (R26^*Ddo*/*Ddo*^), characterized by constitutive *Ddo* overexpression and, thus, embryonic depletion of D‐aspartate in the brain, have demonstrated altered memory abilities (De Rosa et al. 2022). In addition, nuclear magnetic resonance spectroscopy and high‐resolution mass spectrometry revealed that D‐aspartate plays a role in L‐amino acid and lipid metabolism, as well as in the modulation of biochemical pathways governing cellular bioenergetics, brain development, and function (Grimaldi et al. 2021). Among the observed effects, D‐aspartate deficiency is associated with increased levels of choline (precursor of the neurotransmitter acetylcholine) (Jope 1979), glycine (glutamate co‐agonist for NMDAR activity), glucose, and lactate, among others. Concurrently, its depletion leads to a reduction in other molecules, such as L‐glutamine, as we also observed in this study, and to an oscillatory expression pattern of GABA and L‐glutamate during pre‐ and postnatal developmental stages (Grimaldi et al. 2021). Although the mechanisms through which D‐aspartate influences this wide molecular spectrum require further investigation, we propose that persistently low D‐aspartate levels observed in the hippocampus of *mdx* mice may affect both glutamatergic and GABAergic neurotransmission beyond the direct impact that Dp427 and/or Dp71 deficiency have on post‐ and presynaptic dysfunctions of glutamatergic and GABAergic synapses (Vaillend, Ungerer, et al. 1999; Vaillend, Billard, et al. 1999; Vaillend and Billard 2002; Cyrulnik and Hinton 2008; Daoud et al. 2009a, 2009b; Miranda et al. 2011; Helleringer et al. 2018). Additionally, altered D‐aspartate levels may interfere with yet‐to‐be‐explored metabolic pathways in dystrophic mice, potentially affecting neural circuit development.

Supporting this hypothesis, several metabolic abnormalities have been reported in the brains of DMD patients and animal models of the disease. Bresolin and colleagues (Bresolin et al. 1994) were among the first to correlate lower IQ and cognitive impairment in DMD patients with cortical and cerebellar glucose hypometabolism, as evaluated by positron electron micrography. Magnetic resonance spectroscopy has also revealed abnormal energy metabolism, including significantly increased ratios of inorganic phosphate to adenosine triphosphate (Pi/ATP), phosphomonoesters (Pi/PEM), and phosphocreatine (Pi/Pcr) in the brains of DMD boys compared to controls (Tracey et al. 1995). Elevated ratios of choline (Ch)‐containing compounds to Ch/N‐acetyl aspartate have also been observed (Kato et al. 1997; Rae et al. 1998). Similar metabolic disruption has been documented in *mdx* mice, including increased Pi/PCr ratio and reduced total creatine levels compared to controls. The metabolic profile of this mouse model is notably altered, particularly in the hippocampus, cerebellum, and cortex, with substantial differences between the cerebellum and other brain regions, as well as a distinct temporal pattern (Pomeroy et al. 2025).

*mdx* mice also show reduced levels of brain glucose transporters and glucose hypometabolism (Rae et al. 2002; Wallis et al. 2004), decreased lipid and protein peroxidation, reduced catalase activity, and increased superoxide dismutase activity (Comim et al. 2009). Catalase is an antioxidant enzyme situated in the peroxisomes, the organelles in which D‐aspartate is catabolized by the DDO (reviewed in Pollegioni et al. 2021). In this study, *Ddo* mRNA levels in the hippocampus of *mdx* mice were similar to those in wild‐type mice, following the expected expression pattern over time: E18, P10, and 6 to 7 weeks. Peroxisomal functionality has yet to be explored in either DMD patients or *mdx* mice (or other animal models); however, we cannot exclude the possibility that compromised DDO physiological activity may contribute to the D‐aspartate dysmetabolism observed in *mdx* mice.

Increased superoxide dismutase activity has been proposed as a protective mechanism against increased oxidative stress (Comim et al. 2009). However, it may also reflect mitochondrial dysfunction closely linked to dystrophin deletion, as demonstrated in muscle fibers well before the onset of muscle fiber damage (Moore et al. 2020). Indeed, in *mdx* mice, dysregulated intracellular Ca^2+^ levels in hippocampal and cortical neurons (Lopez et al. 2018), altered energetic metabolism (e.g., mitochondrial respiratory chain complexes and creatine kinase activities) (Tuon et al. 2010), and increased activity of the Krebs' cycle enzyme malate dehydrogenase (Comim et al. 2016) have been observed. These findings further contribute to the complex metabolic scenario, in which we suggest decreased D‐aspartate levels may play a role.

A further interesting finding of this study is the reduced level of D‐aspartate observed in the spinal cord of adult *mdx* mice compared to wild type. Kalb and colleagues (Kalb et al. 1992) were among the first to report, through a detailed autoradiographic analysis, that NMDA receptors are highly expressed in the ventral horn of the rat spinal cord shortly after birth, with a progressive decline over the first 4 weeks, coinciding with motoneuron maturation. These results are consistent with earlier studies demonstrating NMDA receptor activity in embryonic and early postnatal motoneurons (Jahr and Yoshioka 1986; Ziskind‐Conhaim 1990), supporting a pivotal role for NMDA‐mediated neurotransmission in motoneuron differentiation and survival (Brenneman et al. 1990). Further studies have confirmed that NMDA receptor activation is essential for the final stages of dendritic growth and morphological maturation of spinal motoneurons (Verhovshek et al. 2005). Taken together, these findings support the hypothesis that D‐aspartate, through NMDA receptor activation, contributes to motoneuron survival, maturation, and the establishment of motor circuitry during early spinal cord development. In light of these data, our observation of reduced D‐aspartate levels in the spinal cord of adult *mdx* mice may reflect a dysregulation of glutamatergic signaling onto motoneurons in the ventral horn, possibly linked to altered sensory–motor connectivity resulting from retrograde signaling triggered by degenerating muscles. This novel observation opens new avenues of investigation into NMDA receptor expression and function in the ventral spinal cord across pre‐ and postnatal stages.

In this study, the hippocampus of *mdx* mice also exhibited nearly significant reductions in D‐serine (*p* = 0.073) and its precursor L‐serine (*p* = 0.053), compared to wild‐type mice. D‐serine is another key D‐amino acid whose synthesis and catabolism are regulated by the enzymes SRR and DAAO, respectively. As with D‐aspartate, we did not detect time‐dependent differences in the levels of expression of these enzymes between *mdx* and wild‐type mice. However, as discussed for D‐aspartate, D‐serine dysmetabolism could result from altered enzymatic activity, rather than changes in enzymatic levels, potentially due to Dp427 deficiency impacting mitochondrial and peroxisomal physiology.

As highlighted in the introduction, one of the most critical aspects of DMD research involves the cognitive, behavioral, and neuropsychiatric challenges faced by young patients. These include autism spectrum disorders, depressive mood, schizophrenia (Caspers Conway et al. 2015; reviewed in De Stefano et al. 2022; Hendriksen et al. 2024), and the occurrence of epileptic seizures in approximately 5.6% of young patients, a rate significantly higher than the 0.5%–1% observed in the general pediatric population (reviewed in De Stefano et al. 2022; Kizek et al. 2024). A large body of studies has linked dysmetabolism of D‐aspartate and D‐serine to an increased risk of developing psychiatric conditions, particularly schizophrenia (reviewed in Errico et al. 2015; de Oliveira Souza et al. 2023). Recent works reported decreased D‐aspartate levels in blood serum (Garofalo et al. 2025) and post‐mortem dorsolateral prefrontal cortex (in about 30% of analyzed samples) (Nuzzo et al. 2017) of schizophrenia patients. This cortical D‐aspartate decrease was paralleled by higher DDO activity in the same region (Nuzzo et al. 2017). Altered D‐aspartate levels have also been described in a patient with severe intellectual disability associated with autism spectrum disorder symptomatology and thought disorders (Lombardo et al. 2022). Similarly, D‐serine dysmetabolism has been identified as a key factor in the development of many neurological disorders, including schizophrenia (Hashimoto et al. 1993; Kumashiro et al. 1995; Ohnuma et al. 2008; Calcia et al. 2012; Cho et al. 2016; El‐Tallawy et al. 2017), autism spectrum disorder (Nuzzo et al. 2020), major depressive disorder (Dong et al. 2018; Lin et al. 2019), and epilepsy (Walrave et al. 2018). According to the literature, the NMDAR, since it binds both D‐aspartate and D‐serine, is considered a major dysfunctional target. However, it is important to remember that in our DMD model, L‐glutamate and L‐glutamine, which are also strategic in the GABA metabolic pathway, are decreased in the hippocampus of embryonic (L‐glutamate) and adult (L‐glutamate and L‐glutamine) *mdx* mice compared to wild type. These findings also suggest a significant role of these amino acids in the pre‐ and postnatal neurodevelopmental alterations in dystrophic subjects, at least at the hippocampal level.

As previously mentioned, our HPLC‐based clinical study on blood serum samples from young DMD patients revealed significant amino acid dysregulation compared to healthy subjects (Garofalo et al. 2025). Notably, we observed lower levels of L‐aspartate, L‐asparagine, D‐serine, L‐glutamine, glycine, and D‐/total serine ratio. Moreover, we identified a negative correlation between serum levels of L‐glutamate and L‐aspartate with creatinine and creatine kinase, two established biomarkers of muscle damage. In addition, a direct correlation between the L‐glutamine/L‐glutamate ratio and fat‐free mass suggested a link between L‐glutamate levels and the L‐glutamine/L‐glutamate ratio, muscle wasting and motor impairment. Interestingly, no significant correlation emerged between these amino acids' changes and the severity of neurological symptoms in the patient's cohort. Comparing this study with the clinical one, no significant differences in serum amino acid levels were detected between the two genotypes. These apparent discrepancies may be explained by several factors. First, changes in free amino acid levels in the brain parenchyma and other nervous system regions of DMD patients may not be readily reflected in serum levels, as neurological dysfunctions are not the primary cause of the disease but rather comorbidities. This contrasts with primary neurological disorders, where changes in brain amino acid levels might be more apparent and detectable using highly sensitive methods applied to tissue homogenates from specific regions. Second, as previously mentioned, the *mdx* mouse model presents a milder form of the disease compared to human DMD patients, as these mice lack only the Dp427 isoform while preserving the expression of other dystrophin isoforms. Although the developmental role of the full‐length dystrophin in the nervous system is crucial, the neurological phenotype of *mdx* mice is less severe than in other models, such as the transgenic *mdx3cv* mice. Similarly, muscle damage in *mdx* mice occurs in waves of degeneration and regeneration, preventing complete muscle fiber degeneration and preserving mobility (Partridge 1997). This stands in stark contrast to the aggressive and progressive muscular degeneration observed in DMD patients, which contributes to alterations in specific serum amino acid levels associated with muscle wasting and motor impairment. In *mdx* mice, the absence of definitive and irreversible muscle degeneration likely leads to a milder manifestation of these metabolic changes, making them less detectable in serum.

Support for the notion that alterations in amino acid levels within specific brain regions do not necessarily mirror those found in blood serum comes from a recent study conducted in an animal model of a distinct neurological condition: the environmental autism spectrum disorder rat model, prenatally exposed to either the endotoxin lipopolysaccharide (LPS), which mimics maternal immune activation, or the antiepileptic drug valproic acid (Di Maio et al. 2025). The study revealed a complex pattern of altered D‐aspartate, D‐serine, and other amino acid levels across various brain regions, including the hippocampus. Remarkably, these neurochemical changes were not reflected in either serum or fecal samples, despite the marked inflammation induced by LPS, strongly suggesting they are driven by specific central metabolic alterations supported by epigenetic and neuroinflammatory mechanisms.

### 4.1 What Underlies the Decrease in Hippocampal D‐ and L‐Amino Acids in *mdx* Mice? Exploring Potential Mechanisms

Summing up our findings, among the brain regions analyzed, the hippocampus of the *mdx* mouse model of DMD appears to be the most affected, showing pronounced reductions in D‐ and L‐aspartate as well as in other amino acids. A first important consideration is that the marked reduction in D‐aspartate occurs at E18, a developmental stage when this D‐amino acid is normally still abundant in the brain. In contrast, this difference diminishes in young adult mice, likely due to the physiological decline of D‐aspartate levels after birth, which may obscure genotype‐specific differences later in life. Notably, in 6‐ to 7‐week‐old *mdx* mice, L‐aspartate is significantly reduced along with key amino acids involved in glutamatergic transmission, such as glutamate and glutamine. These changes are accompanied by an almost significant decrease in D‐ and L‐serine levels.

How can a point mutation in the dystrophin gene lead to such substantial neurochemical imbalance? Several hypotheses may be considered in a scenario like the DMD brain, where subtle yet widespread neuronal and physiological alterations compromise the function of complex neural circuits. The first hypothesis is neurocentric. D‐aspartate is synthesized by neurons, as shown in both prenatal and postnatal stages (Hashimoto et al. 1993; Schell et al. 1997; Sakai et al. 1998; Wolosker et al. 2000). Although some studies suggest that this D‐amino acid may originate from its L‐enantiomer (Wolosker et al. 2000; Long et al. 2002; Errico et al. 2018), a biosynthetic pathway well demonstrated for D‐serine through the activity of serine racemase, the enzyme responsible for converting L‐aspartate into its D‐form in mammals, remains unidentified to date. Several morpho‐functional changes have been reported in the *mdx* hippocampus (reviewed in De Stefano et al. 2022), including a ~34% reduction in pyramidal neurons (Miranda et al. 2016). These data would suggest that lower D‐aspartate levels result from neuronal loss. However, this explanation seems unlikely when considering cell number alone. Miranda's study was conducted on ~2‐month‐old mice, an age corresponding to our own adult group, in which the D‐aspartate decrease is already less evident. On the other hand, there is a lack of data on neuron numbers in CA1 during prenatal or early postnatal stages, when we observed the most significant reductions in D‐aspartate. Since current models suggest that CA1 neuronal loss occurs postnatally, the reduction in D‐aspartate observed at E18 may not be directly attributable to decreased neuron number but rather to the metabolic and functional abnormalities previously highlighted, which may be particularly relevant in the hippocampus compared to other brain regions.

The idea that altered amino acid levels may not be directly related to neuronal loss is supported by findings from other brain regions. For example, a 50% neuronal loss, primarily affecting corticospinal neurons, has been reported in the cortex of *mdx* mice (Sbriccoli et al. 1995; Carretta et al. 2001; Minciacchi et al. 2010; Anderson et al. 2002), but in our study, this does not find a corresponding drop in D‐aspartate or other amino acid levels, either prenatally or postnatally. Moreover, previous work has shown that cervical spinal motoneuron numbers are comparable between *mdx* and wild‐type mice (Sbriccoli et al. 1995); yet in the present study, we report a reduction in D‐aspartate levels in total spinal cord homogenate from adult *mdx* mice compared to wild type. Regarding the cerebellum, Purkinje cell loss and gliosis have only been described in 1 out of 13 human autopsy cases, and no cerebellar neuronal loss has been reported in *mdx* mice, despite high Dp427 expression in wild‐type mouse Purkinje cells. Instead, cerebellar alterations appear to involve synaptic function, which includes a ~60% reduction in inhibitory synaptic responses (Wu et al. 2022), a decrease in the number of GABA_A_ receptor‐containing synapses (Knuesel et al. 1999; Fritschy et al. 2003; Craig and Kang 2007), and reduced Purkinje cell input to cerebellar nuclei, along with impaired synaptic vesicle replenishment and reduced Purkinje cell firing (Kreko‐Pierce and Pugh 2022).

The second hypothesis, which does not exclude the contribution of neuronal functional and metabolic dysfunction, is a more astrocentric perspective. Indeed, a prominent role of astrocytes in DMD pathology has been proposed (Wijekoon et al. 2023). Accordingly, the observed reduction in D‐serine, L‐serine, glutamate, and glutamine in the *mdx* hippocampus points toward astrocytic dysfunction, with significant implications for neuronal support and signaling. In fact, astrocytes are the primary source of these amino acids for neurons (Wolosker and Balu 2020), and we can presume that DMD‐linked astrocyte dysfunction would impact amino acid levels.

Yet astrocyte role goes far beyond metabolic support. This glial population accounts for 20%–40% of glial cells in the human CNS and shows remarkable regional and functional heterogeneity—including perivascular, protoplasmic, and perimeningeal subtypes (reviewed in Verkhratsky et al. 2023). Their intimate relationship with neuronal function is well documented, and astrocytic dysfunction has been implicated in a wide range of conditions, including neuropsychiatric disorders, intellectual disability, autism spectrum disorder, amyotrophic lateral sclerosis, and other neurodegenerative diseases.

A key astrocytic function is the regulation of synaptic activity via tripartite synapses, in which astrocytes physically and functionally interact with synapses to modulate neurotransmission and maintain extracellular homeostasis (Semyanov and Verkhratsky 2021). This is especially relevant at glutamatergic and GABAergic synapses. Astrocytes take up glutamate via excitatory amino acid transporters 1 (EAAT1) and 2 (EAAT2), converting part of it into glutamine via glutamine synthetase (exclusive to astrocytes). This glutamine is then shuttled to presynaptic terminals, reconverted into glutamate, and packaged into vesicles, often alongside D‐aspartate. In GABAergic synapses, GABA reuptake is followed by conversion to alpha‐ketoglutarate and then into glutamate and glutamine. These steps mirror the glutamatergic cycle, with glutamine once again shuttled to presynaptic terminals, where it is used to synthesize GABA. Dysfunction of the glutamate/GABA–glutamine cycle can cause significant synaptic dysregulation and could be implicated in various brain disorders (reviewed in Andersen 2025). Astrocytes also contribute to potassium buffering (via the Na^+^/K^+^ pump and K_ir_4.1), maintain blood–brain barrier integrity, regulate lipid and glucose metabolism, offer metabolic support (e.g., lactate release), modulate redox homeostasis, and perform neuroprotective roles such as reactive astrogliosis (Khakh and Sofroniew 2015; Breslin et al. 2018).

Altogether, the observed alterations in amino acid levels, especially those linked to glutamatergic and D‐amino acid metabolism, strongly support the proposed model, in which astrocytic dysfunction plays a key role in the pathophysiology of DMD‐related brain changes (Wijekoon et al. 2023).

Full‐length dystrophin, as well as the shorter Dp140, Dp71, and Dp40 isoforms, together with DGC components, is expressed by neural stem cells, multipotent self‐renewing cells that give rise to both neurons and glial cells (astrocytes and oligodendrocytes) and are tightly regulated during neuronal and astrocytic differentiation (Romo‐Yáñez et al. 2020). In astrocytes, the heavy dystrophin isoforms (Dp427 and Dp140) are expressed at lower levels than the low‐molecular‐weight isoforms (Dp71 and Dp40). Among these, Dp71 has been described as the key isoform involved in the stabilization of aquaporin‐4 (AQP4) water channels and Kir4.1 (K^+^ inward rectifier 4.1) channels in astrocytes, thereby contributing to water and K^+^ homeostasis. However, even the absence of Dp427 alone, as in *mdx* mice, leads to a range of astrocytic morpho‐functional impairments. These include decreased expression (both at mRNA and protein levels) of Dp71, DGC components, ECM proteins (e.g., laminin, agrin), AQP4, and Kir4.1 channels (Nico et al. 2010; Tetorou et al. 2024); increased blood–brain barrier permeability (Nico et al. 2004; Pelosi et al. 2017); and structural atrophy, likely resulting from cytoskeletal alterations (Nico et al. 2010).

Destabilization of the Dp71‐DGC complex may also compromise the localization and function of EAAT1 and EAAT2 glutamate transporters. Indeed, experimental data in astrocytes derived from DMD patient iPSC‐derived neural stem cells show a reduced ability to remove extracellular glutamate, leading to neurotoxic accumulation that causes neuronal hyperexcitability and damage in co‐culture systems (Patel et al. 2019). This dysfunction is directly linked to dystrophin deficiency, as the use of a read‐through compound (capable of bypassing the premature stop codon in the DMD gene) restored dystrophin expression in DMD astrocytes and rescued the glutamate uptake defect, preventing the associated neurotoxicity (Patel et al. 2019). At the molecular level, the relationship between dystrophin and glutamate homeostasis may be mediated by DGC interactions: both EAAT1 and EAAT2, like AQP4, contain PDZ‐binding motifs that are hypothesized to anchor them to the dystrophin‐syntrophin complex under physiological conditions (Patel et al. 2019). The loss of dystrophin may therefore result in mislocalization or dysregulation of these transporters, contributing to the observed functional impairments.

Beyond their role in ion buffering and neurotransmitter recycling, astrocytes also provide key metabolic support (e.g., lactate release, neurotransmitter precursors), regulate redox balance, and control cerebral blood flow (Patel et al. 2019). Moreover, transcriptomic analyses of human DMD astrocytes reveal dysregulated metabolic pathways, consistent with impaired cellular behavior and altered energy consumption (Lange et al. 2021).

Last, but not least, in vivo, *mdx* mice exhibit reduced hippocampal astrogenesis, which in the mammalian brain normally increases over the lifetime (Stephenson et al. 2025), and reduced brain levels of neurotrophic factors, potentially reflecting insufficient astrocytic metabolic and trophic support (Comim et al. 2019). A direct impact on hippocampal neurogenesis, and possibly astrocyte function, may also result from reduced levels of nitric oxide (NO) produced by skeletal muscle. In healthy muscle tissue, dystrophin anchors the neuronal nitric oxide synthase isoform (nNOS) to the sarcolemma, enabling NO production during muscle contraction (Chang et al. 1996). In *mdx* mice, as in DMD patients, nNOS is drastically reduced, leading to lower systemic NO levels (Gucuyener et al. 2000; Straub et al. 2002). NO is a diffusible signaling molecule and a potent neurogenic modulator (Hindley et al. 1997; Poluha et al. 1997). Consistently, the lack of muscle‐derived NO in *mdx* mice has been associated with impaired adult neurogenesis (Deng et al. 2009), suggesting that skeletal muscle dysfunction in DMD may contribute systemically to central nervous system alterations.

**FIGURE 7:**
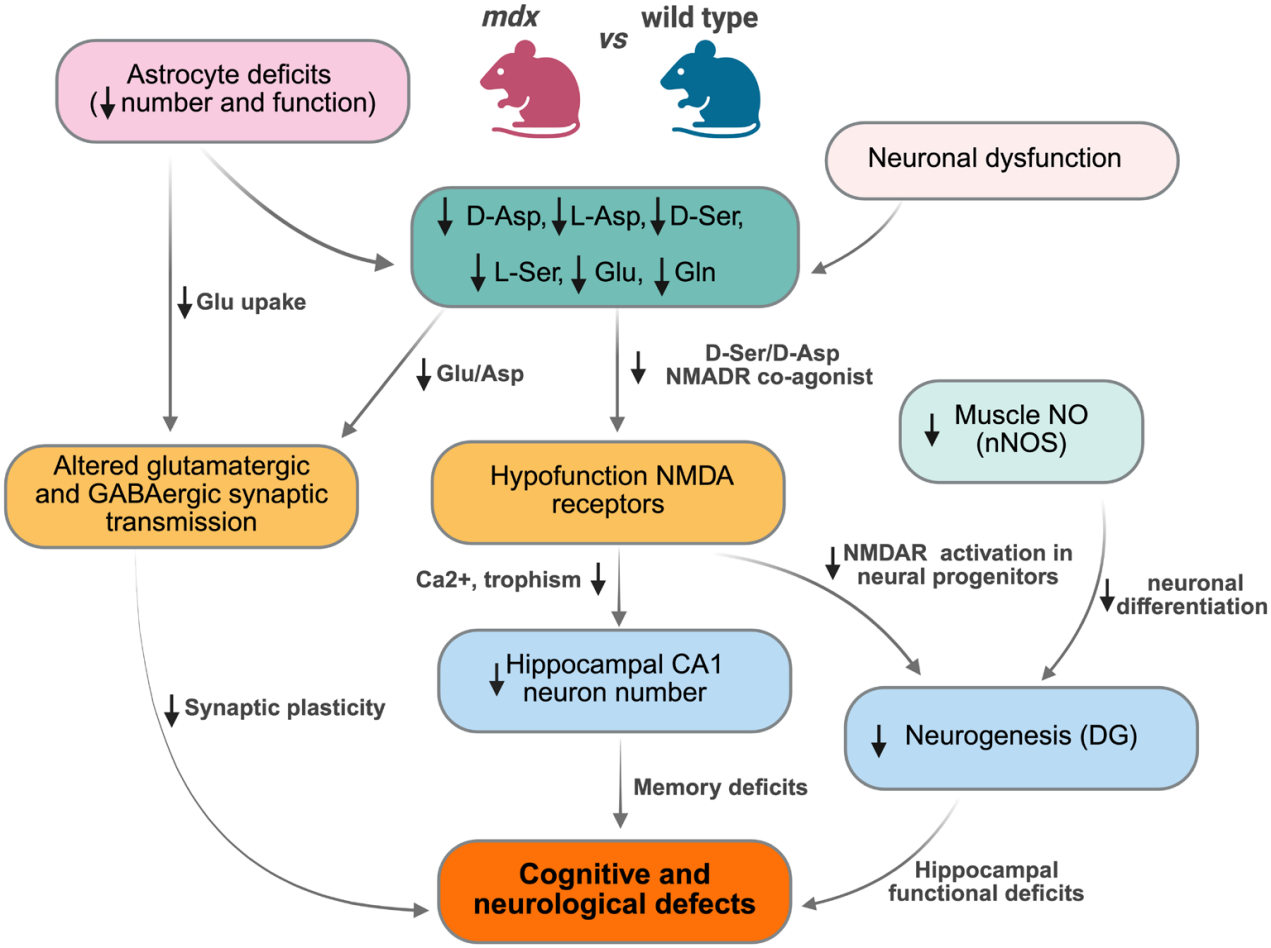
Schematic representation of the hypothesized mechanism linking decreased hippocampal D‐ and L‐amino acid levels to cognitive and neurological alterations. D‐aspartate: D‐Asp; L‐aspartate: L‐Asp; D‐serine: D‐Ser; L‐serine: L‐Ser; glutamate: Glu; glutamine: Gln; nitric oxide: NO; neuronal nitric oxide synthase: NNOS; dentate gyrus: DG; N‐methyl‐D‐aspartate receptors: NMDAR. Image created with BioRender.com (www.biorender.com).

On the whole, these findings indicate that dystrophin deficiency in astrocytes compromises their ability to support neurons across multiple domains: weakened ion homeostasis, impaired clearance of excitatory neurotransmitters, disrupted neuron–glia signaling (via Ca^2+^/NO), and possibly reduced delivery of nutrients and growth factors. A comprehensive scheme of the hypothesized mechanisms linking decreased D‐ and L‐amino acid levels and hippocampal cognitive and neurological alterations is presented in Figure 7.

An open question remains as to why the hippocampus appears to be the most affected region, among those examined in the *mdx* mouse model of DMD, in terms of D‐/L‐amin acid expression. One likely explanation lies in the fact that the hippocampus is among the brain areas with the highest expression of Dp427 and its shorter isoforms (Dp140, Dp71). It is therefore reasonable to assume that it is also the region most vulnerable to dystrophin deficiency, morphologically, anatomically, and functionally. Another, non‐mutually exclusive explanation may relate to the marked morpho‐functional heterogeneity of astrocytes across different brain regions (Morel et al. 2017; Verkhratsky et al. 2023). It is plausible that hippocampal astrocytes, owing to their unique structural and functional properties, are differentially susceptible to dystrophin loss, thereby contributing to region‐specific vulnerability in DMD. Notably, the hippocampus, along with the isocortex and olfactory bulb, also displays the highest glutamatergic synaptic density (i.e., “connections per unit volume”) (Santuy et al. 2020), and therefore, a higher abundance of glutamate receptors. Altogether these factors may contribute to the specific pattern of alterations observed.

### 4.2 Future Perspectives on D‐Aspartate and D−/L‐Amino Acid Alterations in the Hippocampus and Spinal Cord of *mdx* Mice: Overcoming the Limitations of Observational Studies

The primary limitation of any observational study, even when supported by prior clinical evidence or representing the first demonstration of a specific phenotype, is the absence of direct functional validation. In the present work, consistent with our previous clinical study on DMD patients, we confirm a significant dysregulation of D‐ and L‐amino acids involved not only in glutamatergic signaling but also in GABAergic transmission. Focusing on the CNS, our findings clearly indicate that the hippocampus is the brain region most susceptible to non‐muscular alterations in DMD compared to the other areas examined.

The wealth of scientific literature documenting anatomical and functional changes in the hippocampus and other brain regions, affecting both neurons and glial cells (particularly astrocytes), has provided a solid framework for formulating plausible mechanistic interpretations of our findings. This has allowed a spatiotemporal translation of our results across pre‐, peri‐, and postnatal developmental stages and across distinct CNS compartments. Nevertheless, the functional significance of these neurochemical alterations remains to be clarified. This question could be addressed, at least in part, through rescue experiments involving the controlled administration of D‐aspartate.

Indeed, D‐aspartate is known to play a critical role in learning and memory (D'Aniello 2007; Errico et al. 2009; Topo et al. 2010), and decreased levels of D‐aspartate have been revealed in the prefrontal cortex and striatum of schizophrenia patients (Errico et al. 2013; Nuzzo et al. 2017). Several studies have shown that its supplementation via drinking water can enhance physiological cognitive performance (Topo et al. 2010) and alleviate experimentally induced pain and cognitive impairments (Palazzo et al. 2016). A one‐month D‐aspartate treatment in mouse models of neuropathic pain not only reduced pain sensitivity but also mitigated associated symptoms such as cognitive deficits and stereotypical and depressive‐like behaviors, effects paralleled by a reduction of insoluble Aβ1–42 in the hippocampus (D'Aniello et al. 2017). Furthermore, D‐aspartate has been shown to attenuate schizophrenia‐like symptoms induced in mice by amphetamine or MK‐801, partly through the enhancement of long‐term synaptic plasticity (Errico, Rossi, et al. 2008), as well as by phencyclidine administration (Errico et al. 2015), and to promote dendritic growth and arborization (Errico et al. 2014). In a different pathological context, oral D‐aspartate administration in experimental autoimmune encephalomyelitis, a model of multiple sclerosis, delayed the onset of the disease and reduced its severity while also decreasing inflammation and serum interleukin‐6 levels (Afraei et al. 2017). It is important to note, however, that the treatment duration and timing of administration varied considerably across these studies, underscoring that a single therapeutic protocol is unlikely to be optimal for all conditions.

On these premises, it is reasonable to hypothesize that D‐aspartate administration in *mdx* mice could mitigate at least some of the CNS alterations identified, particularly within the hippocampus. However, unlike conditions in which NMDA receptor dysfunction represents one of the primary pathogenic drivers, such as major depressive disorder (Sanacora et al. 2012; Hashimoto 2018) or schizophrenia (Park et al. 2021; Kraguljac et al. 2021), the *mdx* mouse is a natural genetic model of dystrophin deficiency. Despite exhibiting comparable muscle degeneration, its neurological phenotype is milder than that in human DMD. Consequently, a complete reversal of all anatomical and functional abnormalities may be unlikely. Careful optimization of treatment parameters, including timing (e.g., administration to pregnant dams and/or adult mice), route, and dosage, will be crucial to avoid misleading false‐positive or false‐negative outcomes.

Although perhaps less striking, the observed reduction in D‐serine and its biosynthetic precursor L‐serine in the hippocampus of adult *mdx* mice also warrants attention. D‐serine is already used in clinical contexts for major depressive disorder (Sempach et al. 2025) and schizophrenia (Mirhashemi et al. 2025), both conditions linked to NMDA receptor dysfunction, with improvements reported in depressive symptoms and working memory, respectively.

These findings suggest that targeting multiple amino acid pathways, rather than focusing solely on D‐aspartate supplementation, may represent a more effective therapeutic approach for addressing the CNS deficits associated with dystrophin deficiency.

## 5 Conclusions

In conclusion, D‐aspartate, L‐glutamate, L‐glutamine, and D‐serine can rightfully be considered true neuromodulators in the mammalian brain, playing a key role in shaping neural circuitry and influencing essential neurological and cognitive functions. This study provides the first evidence of significant dysregulation of D‐amino acids in the dystrophic *mdx* mouse hippocampus, a region notably affected in DMD. Remarkably, these alterations are already evident at prenatal stages, highlighting a developmental impact of dystrophin deficiency. While further studies are needed to elucidate the precise molecular mechanisms linking these imbalances to the absence of Dp427, a growing body of evidence supports an astrocyte‐centered hypothesis. Why such dysfunction appears particularly pronounced in the hippocampus and not in other brain regions such as the cortex or cerebellum, which are also devoid of Dp427, remains an open question. Altogether, our findings provide a compelling rationale for further investigating D‐aspartate and related amino acids as potential therapeutic candidates to alleviate the neurological comorbidities associated with DMD.

## Author Contributions

**Francesca Mastrostefano:** investigation, data curation, formal analysis. **Martina Garofalo:** investigation, data curation, formal analysis. **Tommaso Nuzzo:** investigation, data curation, formal analysis, writing – review and editing. **Claudio Bruno:** writing – review and editing. **Francesco Errico:** writing – review and editing, conceptualization. **Alessandro Usiello:** conceptualization, data curation, formal analysis, writing – review and editing, writing – original draft. **Maria Egle De Stefano:** conceptualization, data curation, formal analysis, writing – original draft, writing – review and editing, funding acquisition.

## Conflicts of Interest

The authors declare no conflicts of interest.

## Supporting information


**Data S1:** jnc70223‐sup‐0001‐DataS1.pdf.

## Data Availability

The data that support the findings of this study are available from the corresponding author upon reasonable request.
