# Supplementary material for: Central Neurophysiological Alterations in Dystrophic mdx Mice Correlate With Reduced Hippocampal Levels of the Endogenous NMDA Receptor Ligand D‐Aspartate
Source: J Neurochem. 2025 Sep 6;169(9):e70223. doi: 10.1111/jnc.70223 (PMC12413625; doi:10.1111/jnc.70223)
Supplement: Supplementary file 1 — Data S1: jnc70223‐sup‐0001‐DataS1.pdf. [file JNC-169-0-s001.pdf]

Central neurophysiological alterations in dystrophic *mdx* mice correlate with reduced hippocampal levels of the endogenous NMDA receptor ligand D-aspartate

Francesca Mastrostefano[1]*, Martina Garofalo[2,3] *, Tommaso Nuzzo[2,3]*, Claudio Bruno[4,5], Francesco Errico[3,6], Alessandro Usiello[2,3@], Maria Egle De Stefano[1,7@]

[1]Department of Biology and Biotechnologies "Charles Darwin", Sapienza University of Rome, Rome, Italy;
[2]Department of Environmental, Biological and Pharmaceutical Sciences and Technologies, Università degli Studi della Campania "Luigi Vanvitelli", Caserta, Italy;
[3]CEINGE Biotecnologie Avanzate Franco Salvatore, Napoli, Italy;
[4]Centre of Translational and Experimental Myology, IRCCS Istituto Giannina Gaslini, Genoa, Italy
[5]Department of Neurosciences, Rehabilitation, Ophthalmology, Genetics, Maternal and Child Health (DINOGMI), University of Genoa, Genoa, Italy;
[6]Department of Agricultural Sciences, University of Naples "Federico II", Portici, Italy;
[7]Center for Research in Neurobiology "Daniel Bovet", Sapienza University of Rome, Rome, Italy

* Francesca Mastrostefano, Martina Garofalo and Tommaso Nuzzo contributed equally to this work and share first authorship

Supplementary Table 1. List of primers used for the Quantitative Reverse Transcriptase Polymerase Chain Reaction (qRT-PCR)**.** β-actin and Ppp1a are used as the internal references

**Gene**	**Primers Sequence**
Ddo	F- ACC ACC AGT AAT GTA GCG GC<br>R- GGT ACC GGG GTA TCT GCA C
Daao	F- TTT TCT CCC GAC ACC TGG C<br>R- TGA ACG GGG TGA ATC GAT CT
Srr	F- CCC TTG GTA GAT GCA CTG GT<br>R- TCA GCA GCG TAT ACC TTC ACA C
β-actin	F- CTA AGG CCA ACC GTG AAA AG<br>R- ACC AGA GGC ATA CAG GGA CA
Ppp1a	F- GTG GTC TTT GGG AAG GTG AA<br>R- TTA CAG GAC ATT GCG AGC AG

Supplementary Figure 1

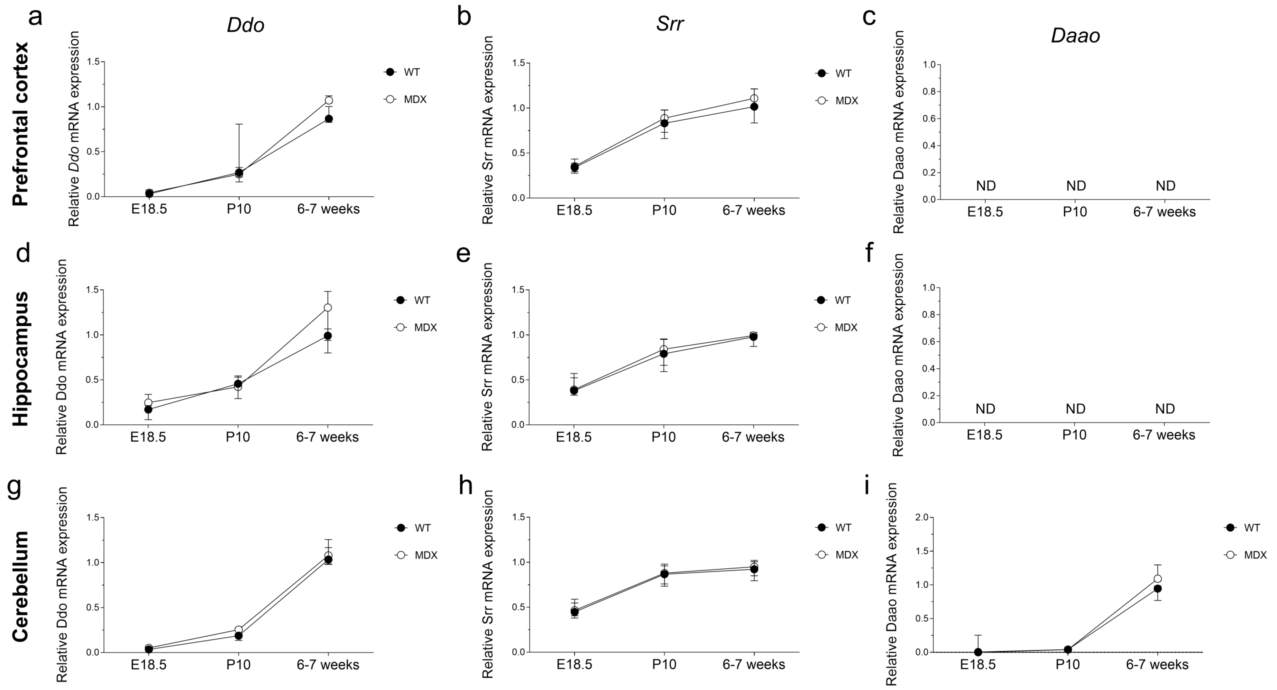

Supplementary Figure 1. qRT-PCR analysis of mRNA levels of *Ddo*, *Daao* and *Srr* in the prefrontal cortex, hippocampus and cerebellum of E18, P10 and 6-7-week-old wild type and *mdx* mice. The mRNA levels of D-aspartate oxidase (*Ddo*), Serine racemase (*Srr*) and D-aminoacid oxidase (*Daao*) vary across pre- to post-natal dates, consistent with their roles at different developmental stages. However, no differences in mRNA levels between the two genotypes are

observed at any of the age considered. Data are expressed as median with interquartile range of relative mRNA expression (n = 6 mice/genotype) and analyzed by two-way ANOVA among age points and genotypes.
